# A Computationally Optimized Broadly Reactive Hemagglutinin and Neuraminidase Vaccine Boosts Antibody-Secreting Cells and Induces a Robust Serological Response, Preventing Lung Damage in a Pre-Immune Model

**DOI:** 10.3390/vaccines12070706

**Published:** 2024-06-24

**Authors:** Pan Ge, Yailin Campos Mota, Robert A. Richardson, Ted M. Ross

**Affiliations:** 1 Center for Vaccines and Immunology, University of Georgia, Athens, GA 30602, USAricharr18@ccf.org (R.A.R.); 2 Department of Infectious Diseases, University of Georgia, Athens, GA 30602, USA; 3 Florida Research and Innovation Center, Cleveland Clinic, Port Saint Lucie, FL 34987, USA; camposy@ccf.org; 4 Department of Infection Biology, Lehner Research Institute, Cleveland Clinic, Cleveland, OH 44195, USA

**Keywords:** influenza, vaccine, antibody-secreting cells (ASCs), serological responses, lung inflammation, hemagglutinin (HA), neuraminidase (NA)

## Abstract

The hemagglutinin (HA) and neuraminidase (NA) surface proteins are the primary and secondary immune targets for most influenza vaccines. In this study, H2, H5, H7, N1, and N2 antigens designed by the computationally optimized broadly reactive antigen (COBRA) methodology were incorporated into an adjuvant-formulated vaccine to assess the protective efficacy and immune response against A/Hong Kong/125/2017 H7N9 virus challenge in pre-immune mice. The elicited antibodies bound to H2, H5, H7, N1, and N2 wild-type antigens; cH6/1 antigens; and cH7/3 antigens, with hemagglutinin inhibition (HAI) activity against broad panels of the H2Nx, H5Nx, and H7Nx influenza strains. Mice vaccinated with the pentavalent COBRA HA/NA vaccine showed little to no weight loss, no clinical signs of diseases, and were protected from mortality when challenged with the lethal H7N9 virus. Virus titers in the lungs of vaccinated mice were lower and cleared more rapidly than in mock-vaccinated mice. Some vaccinated mice showed no detectable lung injury or inflammation. Antibody-secreting cells were significantly increased in COBRA-vaccinated mice, with higher total Ig and H7-specific ASC. Thus, the combination of H2, H5, H7, N1, and N2 COBRA antigens presents a potential for the formulation of a universal influenza virus vaccine.

## 1. Introduction

Influenza viruses are known for their global distribution and high infection rate among high-risk groups, resulting in a strong recommendation for vaccination in this subset of the population [1,2,3]. Some influenza strains can be zoonotic and transmitted to people [4]. A zoonotic influenza virus, which may be antigenically novel to the human population, has pandemic potential if the virus can circulate stably from human to human [5,6]. In nature, influenza viruses circulate continuously among wild bird species and have the potential to evolve into pandemic strains [7]. Unlike seasonal influenza viruses among humans, sporadic human infections with the avian influenza viruses (AIVs) H5N1 and H7N9 can lead to more severe disease outcomes and higher mortality rates than any other subtypes of influenza virus [8,9,10]. The first case of H7N9 AIV in humans was reported in 2013 [11], and more than 1500 people became infected and ~40% of these infections resulted in death [12]. In addition, the infection of domestic poultry with H7 influenza viruses results in dramatic economic losses. In September 2023, more than 54,000 laying hens were affected by an outbreak of H7 highly pathogenic avian influenza (HPAI) in Mozambique [13,14]. Vaccine preparedness and coordination among public health entities will be necessary to prevent future pandemics caused by zoonotic influenza viruses.

Most influenza vaccine platforms use hemagglutinin (HA) and/or neuraminidase (NA) as vaccine antigens to elicit immune responses that will interfere with the HA and NA activity during viral infection [15,16,17,18]. The formulation of a recombinant protein with a potent adjuvant is a promising approach to prevent the severe disease caused by infection [19,20,21,22,23]. Our laboratory has developed a computationally optimized broadly reactive antigen (COBRA) methodology for the design of seasonal and pre-pandemic influenza vaccines. This methodology utilizes multiple rounds of layered consensus sequences to generate influenza virus HA and NA vaccine antigens capable of eliciting broadly reactive antibodies. Z1 is one of the influenza H2 subtype vaccine candidates; 382 unique wild-type H2 sequences from avians, swine, and humans were used for the generation of the COBRA H2 HA vaccines. Z1 was able to confer complete protection and elicit broad immune responses in animal models [24]. IAN8 is a COBRA H5 subtype vaccine candidate; avian and human H5 influenza viruses isolated between 2011 and 2017 were used to generate the COBRA H5 HA vaccines [25]. Q6 is a COBRA H7 vaccine candidate that was generated from avian and human sequences isolated between 2000 and 2018 [26]. N1-I was developed from avian, swine, and human strains isolated between 1990 and 2015 [27]. N2-B was designed from human sequences isolated between 2000 and 2009.

Currently, most preclinical studies evaluate vaccine candidates in immunologically naïve animal models; however, most people have a history of exposure to seasonal influenza viruses [28,29]. In this study, mice were exposed to historical H1N1 and H3N2 influenza strains, resulting in seroconversion to both strains, as previously observed [26]. The pre-immune animal model mimics the pre-existing immune conditions in humans and was used to evaluate the efficacy of COBRA recombinant HA/NA vaccine(s) against H7N9 influenza virus infection in mice.

In this study, a pentavalent formulation of COBRA Z1 (H2), IAN8 (H5), Q6 (H7), N1-I (N1), and N2-B (N2) was tested in a pre-immune mouse model. This is the first time that H2, H5, H7, N1, and N2 COBRA antigens have been used in a pentavalent vaccine formulation in a pre-immune setting. The pentavalent COBRA HA and NA vaccine elicits HAI antibody activity against broad panels of H2Nx, H5Nx, and H7Nx (“x” represents any subtype of NA on the viral surface in the range of 1~11) subtypes and confers protection against lethal H7 viral challenge. Compared to pre-immunized but unvaccinated mice, vaccinated mice had less detectable virus in their lungs at 3, 4, or 6 days post challenge with almost no lung injury. In addition, the pentavalent COBRA vaccine elicited IgG antibodies toward group 1 stem domain and antibodies toward group 2 stem domain, and our laboratory has previously demonstrated that the elicitation of group 1 stem antibodies can induce antibody-dependent cell-mediated cytotoxicity (ADCC) activity by vaccination with another antigen designed using the COBRA methodology [30]. Collectively, these COBRA antigens elicited broad seroprotective antibodies against panels of antigenically distinct H2, H5, and H7 and N1 and N2 virus strains, making the pentavalent COBRA vaccine a promising pre-pandemic vaccine against the H7N9 virus.

## 2. Materials and Methods

### 2.1. Design of COBRA HA and NA Proteins

Influenza HA and NA glycoproteins representing the H2, H5, H7, N1, and N2 subtypes were designed using the computationally optimized broadly reactive antigen (COBRA) methodology [24,25,26,27]. Briefly, wild-type full-length HA and NA protein sequences were downloaded from the Global Initiative on Sharing Avian Influenza Data (GISAID) EpiFlu and National Center for Biotechnology Information (NCBI) Databases as previously described [24,25,26,27,30]. The final consensus sequences were generated after multi-layered consensus sequence building and were considered as the vaccine candidates for each subtype. The soluble HA sequences located at residues 1 to 566 and the NA sequences located at residues 1 to 470 were synthesized and expressed. The stock concentration of the purified proteins was determined by a bicinchoninic acid (BCA) assay. Z1, IAN8, and Q6 represent the COBRA HA vaccine candidates for H2, H5, and H7, respectively. N1-I and N2-B represent the COBRA NA vaccine candidates for N1 and N2, respectively [24,25,26,27].

### 2.2. Production of Virus-like Particle

The HA sequences from wild-type isolates were expressed on the surface of virus-like particles (VLPs). The full-length H2, H5, and H7 HA sequences were first expressed in the pTR300 vector. The human embryonic kidney (HEK) 293T cells were cultured at 37 °C to 75~90% confluence, and then transfected using a Lipofectamine^TM^ 3000 kit (Thermo Fisher Scientific, Waltham, MA, USA). The corresponding HA plasmid was transiently transfected, as well as the human immunodeficiency virus-1 (HIV-1) Gag plasmid, and mismatched NA (NA genes from 1918 H1N1 virus for H2 VLPs, NA genes from A/Thailand/1(KAN-1)/2004 (H5N1) for the H5 and H7 VLPs. The cells were incubated at 37 °C for 3 days, the supernatant was collected and centrifuged. The supernatant was then filtered and purified by ultracentrifugation (through 20% glycerol at 27,000× *g*) for at least 4 h at 4 °C. Pellets from 9 T150 flasks were resuspended with 500 μL phosphate-buffered saline (PBS) and stockpiled at −80 °C.

### 2.3. Pre-Immunization, Vaccinations, and Infections

**Figure 1:**
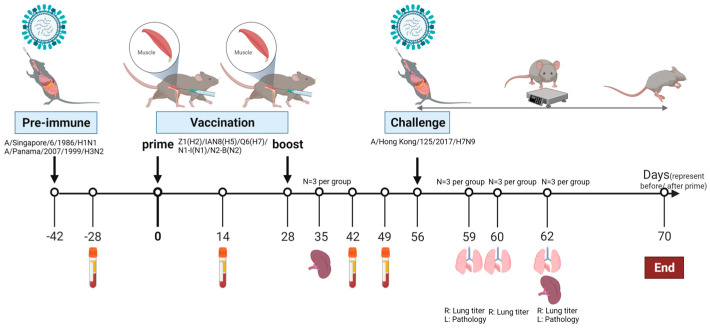
Study design. Schematic diagram of the vaccination regime. Two-month-old female DBA2/J mice were infected with an equal amount of A/Singapore/6/1986/H1N1 (or Sing/86) and A/Panama/2007/1999/H3N2 (or Pan/99) viruses simultaneously or were mock-pre-immunized with PBS. Mice were injected with a pentavalent COBRA vaccine twice intramuscularly or mock-vaccinated with PBS 42 days after pre-immunization. Four weeks after boost vaccination, all the mice were challenged with the lethal A/Hong Kong/125/2017/H7N9 virus. All mice were weighed and checked for survival and clinical symptoms for 14 days. Spleen samples were collected one week after the boost and six days after the H7N9 infection. Blood was collected two weeks after pre-immunization and the boost vaccination. On days 59, 60, and 62, lung samples were collected for viral titration (right lobes) and pathology evaluation (left lobe).

Animal procedures, including intranasal infection, bleeding, sampling, and intramuscular vaccination were approved by the Cleveland Clinic Florida Research and Innovation Center IACUC #2935. All animal studies were performed with female DBA2/J mice ages at approximately 2 months of age (The Jackson Laboratory in Bar Harbor, ME, USA). Thirty-eight mice were randomly divided into 2 groups: the COBRA and mock groups (n = 19). All the mice were simultaneously infected intranasally with the same amounts of A/Singapore/6/1986 (H1N1) and A/Panama/2007/1999 (H3N2) viruses at the same time, at a final concentration of 5 × 10^6^ PFU/50 μL in PBS on day −42 (Figure 1). Half of the mice were vaccinated intramuscularly with the soluble COBRA antigens H2, H5, H7, N1, and N2 on day 0 and day 28. The pentavalent mixture was prepared at 1 μg each with PBS and formulated with AddaVax^TM^ at a 1:1 ratio (InvivoGen, San Diego, CA, USA). Mice in the mock group were injected with 50 μL PBS at six and ten weeks after pre-immunization. Mice in both groups were challenged with 2500 PFU of the A/Hong Kong/125/2017/H7N9 virus four weeks after the boost vaccination.

Blood was collected on days −42, −28, 14, and 42. On day 35 and day 62 post prime, 3 mice in both groups were sacrificed and their spleens were collected. Mice were weighed every day and monitored twice for the first 5 days and once for the remaining 10 days for clinical symptoms, such as lethargy, response to stimuli, and shortness of breath, etc. On days 59 (3 days post infection or 3DPI), 60 (4DPI), and 62 (6DPI), lungs were collected from 3 mice per group for virus titration and pathological evaluation (Figure 1). All mice that lost more than 25% of their initial body weight or exhibited severe symptoms were humanely euthanized when they reached the cut-off, which was a clinical score of at least 3. Survival data are presented daily as the percentage of mice that did not reach the cut-off relative to the initial number of mice.

### 2.4. Enzyme-Linked Immunosorbent Assay (ELISA)

ELISA was utilized to measure the IgG binding to the wild-type HA, NA, and chimeric HA proteins as previously described [26]. Briefly, 96-well Immulon 4 HBX flat-bottom microtiter plates (Thermo Fisher, Waltham, MA, USA) were coated with 10 μg of soluble protein from WT rHA (H2 HA A/Mallard/Wisconsin/08OS2844/2008 or MA/WI/08, H5 HA A/Sichuan/26221/2014 or SC/14, H7 HA A/Anhui/1/2013 or AH/13), rNA (N1 NA A/Vietnam/1203/2004 or VN/04, N2 NA A/Switzerland/9715293/2013 or SW/13) or chimeric rHA (cH6/1 and cH7/3) at 4 °C for overnight. Plates were decanted and blocked with 1% bovine serum albumin (BSA) and 0.13% bovine gelatin in PBST solution for 90 min at 37 °C. After the blocking step, mouse serum diluted to 1:500 was serially diluted three-fold to a final dilution of 1:364,500 in blocking buffer, and was added then incubated for 90 min at 37 °C or overnight at 4 °C. After washing five times with PBST, 100 µL of 1:4000 goat anti-mouse IgG, IgG1, IgG2a, or IgG2b horseradish peroxidase (HRP)-conjugated secondary antibodies (Southern Biotech, Birmingham, AL, USA) was added to each well, and stored in a humidified chamber at 37 °C for 90 min. After washing five times in PBST, 100 μL of 0.1% 2,2′-azino-bis (3-ethylbenzothiaozoline-6–sulphonic acid; ABTS: McIlvaine Solution at 1:10) buffer containing 0.05% H_2_O_2_ was added and incubated at 37 °C for approximately 15 min. Then, 50 μL of 1% sodium dodecyl sulfate (SDS) buffer was added to each well to stop the color development and the plates were read at a wavelength of 414 nm using a PowerWaveXS plate reader (Biotek, Winooski, VT, USA). The endpoint dilution was determined as the last dilution higher than three times that of the blank.

### 2.5. Hemagglutination Inhibition (HAI) Assay

HAI assays were performed to determine the antibodies that inhibited the binding of influenza viruses to sialic acid residues on red blood cells. Serum samples extracted from blood collected 14 days after pre-immunization and boost vaccination were diluted 1:10 with receptor-destroying enzyme (RDE) (a supernatant of Vibrio cholerae serovar Ogawa strain 558; Denka Seiken, Co., Tokyo, Japan) and treated by incubation for 16 h at 37 °C, then transferred to a 56 °C water bath for 1 h. In the v-bottom 96-well microtiter plates, the RDE-treated mixture was two-fold serially diluted from the first column. A total of 25 µL of VLP or influenza virus diluted in PBS was added to the plate to a total of 8 hemagglutination units (HAU)/50 μL. The mixture was then incubated at room temperature (RT) for 20 min and 0.8% turkey red blood cells (TRBCs) were added at a volume of 50 μL, or they were incubated at RT for 30 min, and 1% horse red blood cells (HRBCs) were added. The plates were allowed to incubate with TRBCs (for the H2 and H7 strains) for 30 min or with HRBCs (for the H5 strain) for 1 h. The HAI titers were calculated as the reciprocal dilution of the last well that contained non-agglutinated RBCs.

### 2.6. Lung Viral Titers

Four lung lobes from the right side of both groups of mice were harvested at 3, 4, and 6DPI for virus titration. Briefly, lung samples (n = 3 per time point, per group) were collected and stored at −80 °C. Lung lobes were weighed prior to processing. DMEM (Thermo Fisher, Waltham, MA, USA) containing 1% penicillin–streptomycin (DMEM+P/S) was added ten-fold to thaw and homogenize the lung tissues. Lung homogenates were two-fold serially diluted with DMEM+P/S in a 96-well V-bottom plate first, and then 100 μL of diluted homogenates was transferred to 6-well plates that were overlaid onto a confluent Madin–Darby Canine Kidney (MDCK) cell monolayer for 1 h. The cells were washed two to three times with DMEM+P/S, then covered with 2–3 mL 0.8% agarose plus 2 × Minimum Essential Medium (MEM) overlay containing 1 μg/mL TPCK trypsin. The cells were incubated at 37 °C for 3 days. After this time, the overlay was removed, and 10% formalin was added to each and incubated for 10 min at room temperature. Finally, the monolayer was stained with 1% crystal violet (Thermo Fisher, Waltham, MA, USA) for 15 min. The results were expressed as plaque-forming units (PFUs) per gram of lung tissue.

### 2.7. Lung Histopathology Assessment

The left lung lobes from each group were harvested on days 59 and 62 and inflated with 10% formalin for at least one week. Lung tissues were transferred to PBS and stored at 4 °C overnight. Lung tissues were sectioned and stretched in a 40 °C water bath for less than 1 min, dehydrated through graded ethanol series, infiltrated with xylene, and embedded in paraffin blocks. Paraffin-embedded tissues were sectioned at 5 μm with a microtome, deparaffinized and rehydrated in ethanol, stained with hematoxylin and eosin (H & E), and mounted with coverslips. Inflammatory cell infiltration and lung injury were assessed.

### 2.8. Fluorospot Assay

Antibody-secreting cells (ASCs) were enumerated in mouse spleens collected 7 days after the boost vaccination and 6 days after infection. Prior to antigen coating, the plates were activated with 15 μL/well of 70% ethanol and washed twice with PBS, then 80 μL of A/Guangdong/17SF003/2016/H7N9 rHA or A/Sichuan/26221/2014/H5N6 rHA proteins, anti-Igκ/λ capture antibody, or BSA were coated directly into the wells at 25 μL/mL in PBS, and the plates were placed in a humidified chamber at 4 °C overnight. The plates were washed with 150 μL PBS and then incubated with 150 μL/well of B cell medium (BCM) at RT for 1 h. The splenocytes were two-fold serially diluted in duplicate, starting at 1.5 × 10^5^ live cells/well in plates coated with rHA, and starting at 1 × 10^5^ live cells/well in plates coated with BSA or anti-Igκ/λ capture antibody. The plates were incubated at 37 °C and 5% CO_2_ for 16–18 h. Plate-bound Ig spot-forming units (SFUs) were reflected. The plates were washed with PBST and IgG1-, IgG2a-, and IgG2b-specific reagents were added at a volume of 80 μL/well and incubated at room temperature in the dark for 2 h. The plates were washed with PBST, and a tertiary solution was prepared and added at a volume of 80 μL/well and incubated at room temperature in the dark for 1 h. The plates were washed 4 times with distilled water and then the excess water was vacuum-dried. All reagents were included in the mouse IgG1/IgG2a/IgG2b Three-color ImmunoSpot^®^ kit (CTL, Shaker Heights, OH, USA). The plates were completely air-dried and scanned on an ImmunoSpot^®^ S6 Ultimate Analyzer. The SFUs were counted using the Basic Count mode of the CTL ImmunoSpot SC Studio (version 1.6.2, Shaker Heights, OH, USA). The count number from each well was averaged, then multiplied by the dilution factor and finally divided by the number of live cells coated on each well.

### 2.9. Quantification and Statistical Analysis

Data are presented as absolute mean ± standard error of the mean (SEM). Unpaired t-test, one-way ANOVA, and two-way ANOVA were used to analyze the statistical differences in different scenarios. A “*p*” value less than 0.05 was defined as statistically significant (*, *p* < 0.05; **, *p* < 0.01; ***, *p* < 0.001; ****, *p* < 0.0001; ns, not significant). All statistical analyses were performed with GraphPad Prism 10 software (GraphPad, San Diego, CA, USA).

## 3. Results

### 3.1. Vaccination and Infection of Mice

Female DBA/2J mice (n = 38) were infected simultaneously with a mixture of historical H1N1 and H3N2 influenza viruses and subsequently seroconverted to both strains. Mice were vaccinated intramuscularly and boosted at day 28 with a pentavalent mixture of COBRA HA and NA proteins, or were mock-vaccinated with PBS (Figure 1).

### 3.2. In Vivo Protection Efficacy Evaluation

**Figure 2:**
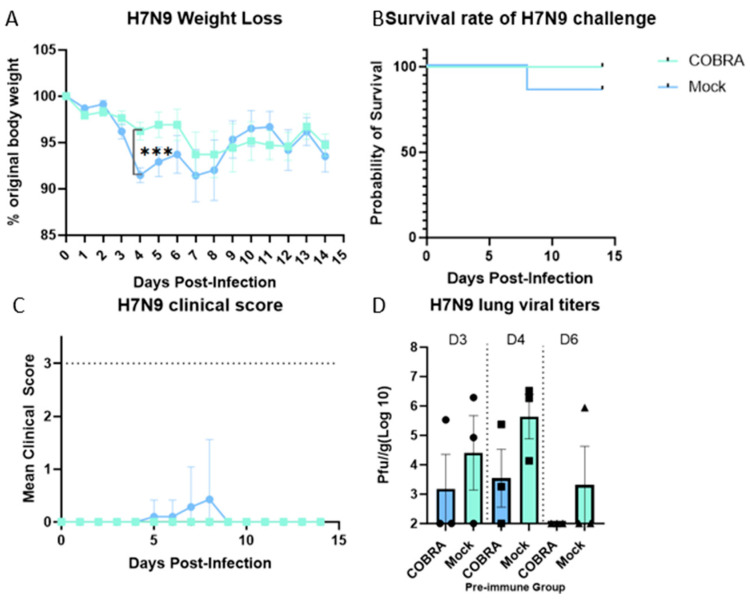
COBRA HA/NA vaccination elicited effective protection against H7N9 challenge. DBA 2/J mice were challenged by lethal dose of A/Hong Kong/125/2017/H7N9 virus at day 56. Mice were monitored for 14 days for weight change and clinical symptoms. Weight loss (**A**), survival rate (**B**), clinical score (**C**), and lung viral titers (**D**) 3, 4, and 6 days post infection were measured. Data shown are mean ± SEM, n = 7 per group for weight change and survival, and n = 3 per group for 3, 4, and 6 days post-challenge sampling; *** *p* < 0.001 (one-way ANOVA with Tukey’s post-test).

All mice were challenged intranasally with the A/Hong Kong/125/2017 (H7N9) virus (2500 PFU/50 μL) at day 56. Mice vaccinated twice with the pentavalent COBRA HA/NA vaccine lost an average ~7% of their original weight by day 7 post infection following HK/17 challenge (Figure 2A). All mice survived the challenge with no detectable clinical signs (Figure 2B,C). Mock-vaccinated mice lost significantly more body weight by 4 days post infection; one mouse succumbed by 8 days post infection on which clinical scores peaked too (Figure 2A–C). Mice vaccinated with the COBRA HA/NA vaccines had statistically low viral lung titers (~10^3^ pfu/g) at 3–4 days post infection with no detectable viral lung titers in 50% of the mice (Figure 2D). In contrast, ~83% of mock-vaccinated mice had a detectable viral lung titer that was on average two logs higher than COBRA HA/NA-vaccinated mice (~10^5^ pfu/g). There were no detected viral lung titers on day 6 post-infection in COBRA HA/NA-vaccinated mice (Figure 2D), whereas one mock-vaccinated mouse still had a viral titer of ~10^6^ pfu/g on day 6 post infection.

### 3.3. Assessment of Lung Injury and Inflammation

**Figure 3:**
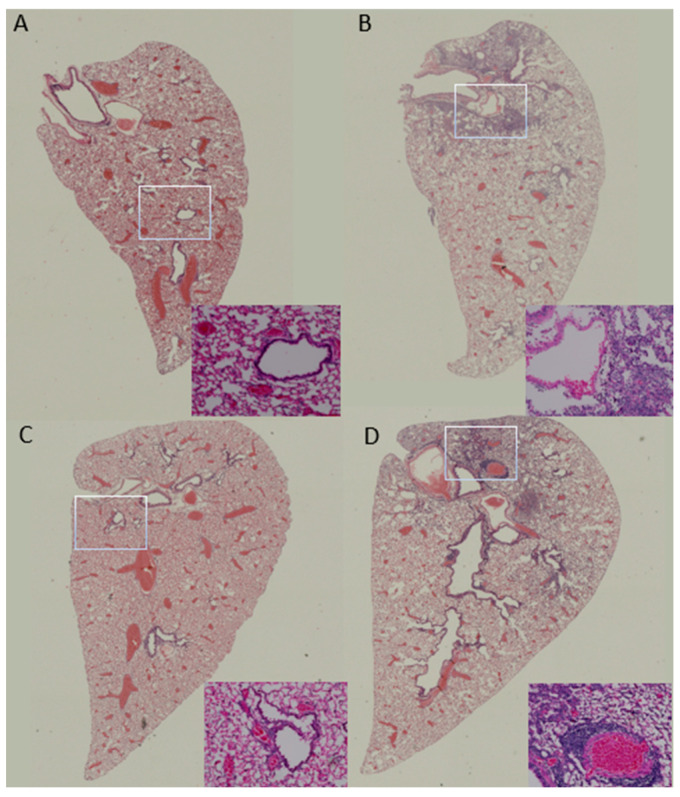
Lung pathology in mice after infection with A/Hong Kong/125/2017/H7N9. The left lung lobe was perfused with 10% formalin for at least 24 h before pathological evaluation. H&E staining was performed on 5 μm lung tissue slides to determine lung injury and inflammatory cell infiltration. Representative images from the (**A**,**C**) COBRA and (**B**,**D**) mock groups were taken from lungs collected at 3DPI (**A**,**B**) and 6DPI (**C**,**D**). The images at the bottom right of each panel were digitally scanned at 20X magnification. Rectangular frames indicate the area of the lung sample image in the corner that was extracted.

There was no detectable lung injury or inflammatory cell infiltration observed from formalin-fixed lungs collected at either 3 or 6 days post infection from COBRA HA/NA-vaccinated mice (Figure 3A,C). In contrast, pre-immune mock-vaccinated mice had apparent lung damage on both days with alveolar septal thickening and condensed neutrophil penetration into the air space, especially located at the connection to the respiratory tract and large blood vessels (Figure 3B,D).

### 3.4. Elicitation of HAI Antibodies by COBRA HA/NA Vaccines

Pre-immune mice vaccinated with COBRA HA/NA proteins had antibodies with HAI activity that recognized a broad number of H2Nx, H5Nx, and HNx7 influenza strains (Table 1). Mice vaccinated twice with COBRA HA/NA vaccines had low HAI activity against the panel of H2Nx viruses (Table 1A). On average, the HAI titers were less than 1:40 against these H2 strains, except the Mal/01 strain that had an average titer of 1:80 (Table 1A). Similar results were observed in collected sera against the panel of H7Nx viruses (Table 1C). Fewer than 50% of the mice had a detectable HAI titer against the viruses in these panels. These same sera had higher HAI activity against a panel of H5Nx influenza viruses (Table 1B). In total, 14/16 and 12/16 had HAI activities against the VN/04 and WS/05 strains, respectively, with an average HAI titer of ~1:40. HAI activity was detected against all six H5Nx strains; however, activity was weakest only against SC/14. Overall, there was a wide range of HAI titers against all the viral strains in each panel that ranged from a titer of 1:10 to, in one instance, 1:2048 (Table 1). No detectable HAI titer was observed in the mock-vaccinated mice.

### 3.5. Elicitation of IgG Antibodies by COBRA HA/NA Vaccines

**Figure 4:**
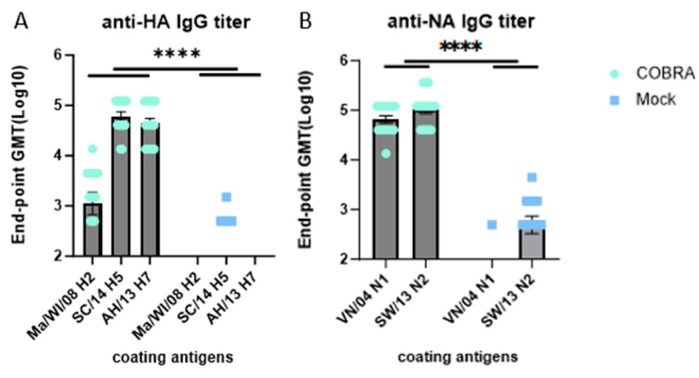
Wild-type HA- and NA-specific IgG antibody levels in COBRA HA- and NA-vaccinated mice 2 weeks (D42) after the boost vaccination. Serum collected from DBA2/J mice was used to determine the endpoint titer against A/Mallard/Wisconsin/08OS2844/2008 or anti-Ma/WI/08 H2, anti-A/Sichuan/26221/2014 or anti-SC/14 H5, and anti-A/Anhui/1/2013 or anti-AH/13 H7 HA in (**A**), and anti-A/Vietnam/1203/2004 or anti-VN/04 and anti-A/Switzerland/9715293/2013 or anti-SW/13 NA in (**B**). Data shown are mean ± SEM, n = 16 per group in each experiment; **** *p* < 0.0001 (one-way ANOVA with Tukey’s post-test).

Antibodies elicited by pre-immune viral infections did not bind to any of the recombinant HA or NA proteins following infection. To determine if COBRA HA/NA vaccination elicited high-titer IgG antibodies against these wild-type proteins, sera collected following the second vaccination were examined by ELISA (Figure 4). All mice vaccinated with COBRA HA/NA antigens had antibodies that bound to the three H2, H5, and H7 HA and the two N1 and N2 NA proteins (Figure 4).

### 3.6. Elicitation of HA Stem-Directed Antibodies by COBRA HA/NA Vaccines

**Figure 5:**
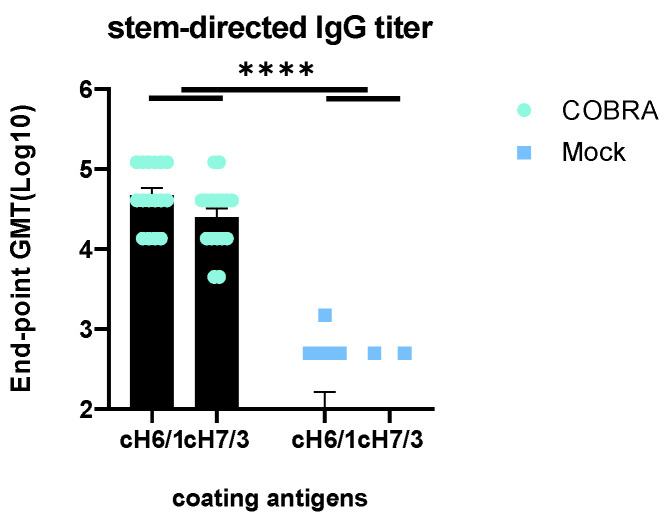
Serological anti-cH6/1 and anti-cH7/3 IgG levels on day 42. Endpoint titers were measured against cH6/1 and cH7/3. Chimeric HA cH6/1 consists of head from A/Mallard/Sweden/81/2002(H6N2) and stalk from A/California/07/2009(H1N1pdm); cH7/3 consists of head from A/Anhui/1/2013(H7N9) and stalk from A/Texas/50/2012(H3N2). Data shown are mean ± SEM, n = 16 per group in each experiment; **** *p* < 0.0001 (one-way ANOVA with Turkey’s post-test).

Mock-vaccinated mice with pre-existing anti-influenza immunity had low antibody titers (<10^3^ endpoint dilution) against both the group 1 and 2 HA stem portions of HA (Figure 5), whereas pre-immune mice vaccinated with COBRA HA/NA vaccines induced significantly higher HA stem-binding antibodies to both the group 1 and 2 stem regions of HA. No significant difference was observed between these two chimeric proteins in the Pre-immune COBRA group. Significantly higher binding to cH6/1 than cH7/3 was observed in the Pre-immune Mock-vaccinated group (*p* = 0.0073), with 2/16 mice showing baseline binding activity to cH7/3 and 9/16 showing binding activity to cH6/1 (Figure 5).

### 3.7. Th1/Th2 Antibodies Predominance and Trend

**Figure 6:**
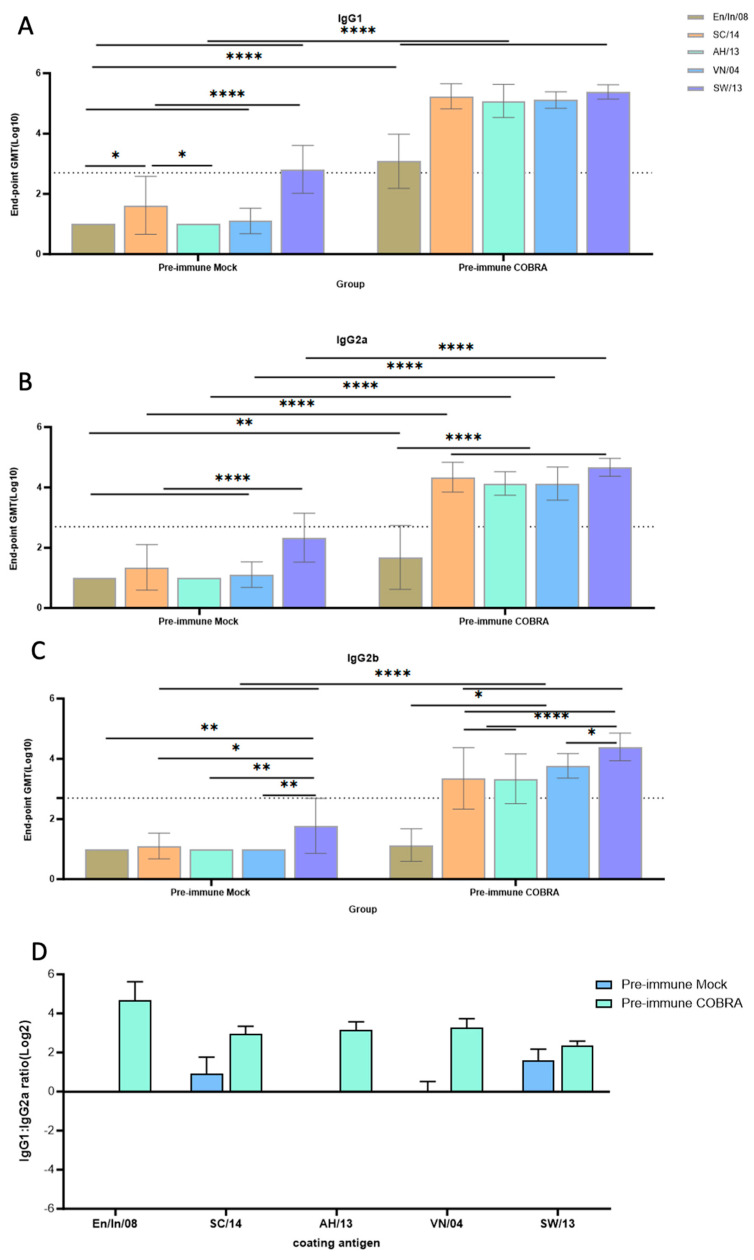
IgG1, IgG2a, and IgG2b levels and IgG1/IgG2a ratio. Anti-Ma/WI/08 H2, anti-SC/14 H5, anti-AH/13 H7, anti-VN/04 N1, and anti-SW/13 N2 IgG1 (**A**), IgG2a (**B**), and IgG2b (**C**) endpoint titers were measured in mouse serum collected on D42. The IgG1:IgG2a ratio (**D**) was used to determine the Th1/Th2 bias. The data shown are mean ± SEM, n = 16 per group in each experiment. The dash line represents the limit of detection (LOD) (serum dilution at 1:500). * *p* <0.05, ** *p* < 0.01, **** *p* < 0.0001.

The IgG subclass profile in each mouse group was assessed and the IgG1/IgG2a ratio was calculated (Figure 6). These pre-immune mock-vaccinated mice had low-to-undetectable IgG1-biased antibodies against these HA and NA proteins (Figure 6). The levels of all IgG subclasses were significantly lower in the Pre-immune Mock group compared to the Pre-immune COBRA group (Figure 6A–C). The IgG1/IgG2a ratio (in Log_2_) was higher than two against each HA/NA protein tested in the Pre-immune COBRA group, which is strong evidence of a Th2-polarized immune response. This ratio ranged from 0.5 to 2 against each tested antigen in the Pre-immune Mock group (Figure 6D).

### 3.8. COBRA HA/NA Vaccines Elicit Robust Antibody-Secreting Cells (ASCs)

**Figure 7:**
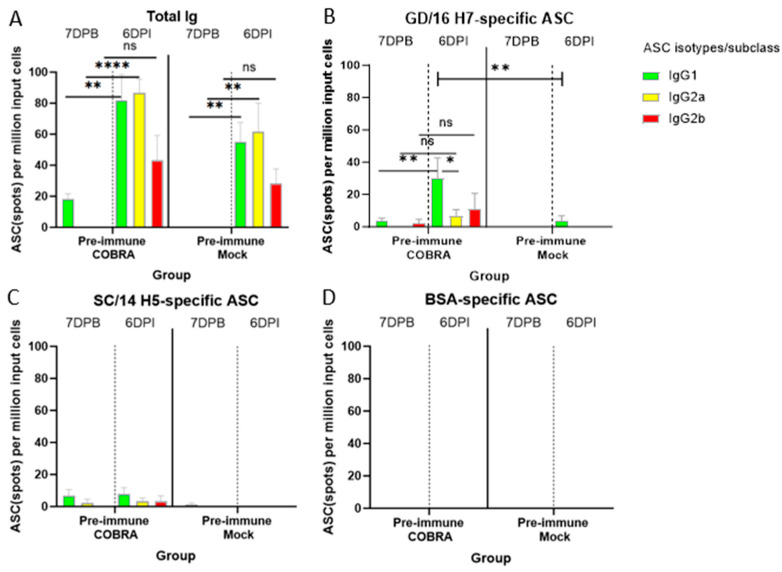
Enumeration of total (**A**), GD/16 H7 (**B**), and SC/14 H5 (**C**) Ag-specific ASCs from splenocytes collected at 7 days after the boost vaccination (7DPB) and 6 days after H7N9 infection (6DPI). BSA was measured as an irrelevant antigen for ASC background reactivity purposes (**D**). A/Guangdong/17SF003/2016/H7N9 rHA or GD/16 H7 and A/Sichuan/26221/2014/H5N6 rHA or SC/14 H5 were used for Ag-specific plate coating. The data shown are mean ± SEM using splenocytes from different mouse groups. * means *p* < 0.05, ** *p* < 0.01, **** *p* < 0.0001, “ns” not significant (two-way ANOVA with Tukey’s post-test).

The splenocytes collected at 7DPB and 6DPI were utilized to determine the magnitude and IgG subclass distribution of the ASCs in different mouse groups. The total IgG ASC response was mainly IgG1, followed by IgG2a and IgG2b at both time points before and after challenge (Figure 7A–C). Before the challenge, a low number of IgG1 ASCs were detected in the Pre-immune COBRA group (Figure 7A). After the challenge, both IgG1 and IgG2a ASCs were significantly increased in both groups. IgG2b ASCs were also increased with no significant difference in both groups (Figure 7A). ASCs from Pre-immune COBRA mice mainly secreted GD/16 H7-specific IgG1, which increased significantly after the HK/17 H7N9 challenge and was significantly higher than that of the IgG1 ASC response in the Pre-immune Mock group after the challenge. Additionally, the GD/16 H7-specific IgG1-specific ASC response was significantly higher than that of the IgG2a ASC response. GD/16 H7-specific IgG2a and IgG2b ASCs increased with no significant difference detected (Figure 7B). Low levels of SC/14 H5-specific ASCs were detected either before or after the challenge. No SC/14 H5-specific ASCs were detected in the Pre-immune Mock group (Figure 7C). BSA-specific ASCs were measured to confirm the absence of irrelevant antigen binding detection across all groups (Figure 7D).

## 4. Discussion

The influenza virus surface glycoproteins HA and NA are targets of the immune system in order to protect the host from influenza virus infection. Antibodies directed against the HA head can interfere with sialic acid receptor binding on the host cell surface, thereby preventing infection. Antibodies directed against the HA stalk/stem interrupt the fusion of the viral and endosomal membranes, preventing the release of the viral genome into the infected cell and disrupting viral replication. Antibodies targeting NA can prevent new virions budding from infected host cells, thereby limiting spread to the neighboring cells in the respiratory mucosa [31,32]. The goal of developing a broadly reactive pre-pandemic influenza vaccine is to provide protection against influenza viruses with pandemic potential by eliciting a broad spectrum of immune responses. Influenza HA proteins, in particular the globular head domain, are highly variable, and the COBRA methodology was used to generate vaccine candidates capable of covering multiple epitopes to address the antigenic diversity and to preserve the conserved epitopes to elicit protection against previous and future strains. Influenza H7N9 viruses infected more than 1500 people in 2013–2017 and caused a mortality rate of an approximately 40% [12]. They are still circulating in animal reservoirs. In 2019, one human infection with the H7N9 HPAI virus was reported in China. The same HPAI H7N9 viruses were detected in the poultry market around the human case. Sixteen amino acid substitutions were found in the new human strain A/gansu/23277/2019, which showed low cross-reactivity with previous vaccine strains, suggesting that H7N9 viruses are still evolving and have the ability to infect humans [33].

A/Hong Kong/125/2017 is a low-pathogenic avian influenza (LPAI) H7N9 virus strain from the Yangtze River Delta (YRD) lineage. In March 2017, A/Hong Kong/125/2017 was recommended by the WHO as a candidate vaccine virus (CVV) in response to the potential pandemic risk posed by the fifth wave in which HPAI was identified, accounting for 3% of the infections during this wave [34,35]. A dose of 2500 PFU/50 μL of this strain was confirmed to be 100% lethal in female naïve DBA2/J mice. The pre-existing immunity conferred by H1N1 and H3N2 infections provided some protection in the mock-vaccinated mice (Figure 2). In pre-immune but mock-vaccinated mice, weight loss increased to approximately 9% on day 4, and clinical scores peaked on day 8, with one mouse reaching the critical endpoint on the same day (Figure 2A–C). Lung viral titer showed no significant difference between the two groups at 3, 4, or 6 DPI, although the challenge caused lung pathology and inflammation as depicted in the histopathology images, and the virus was cleared by 6 DPI in the COBRA-vaccinated group (Figure 2D and Figure 3). A wide range of HA head-neutralizing antibodies were measured in the Pre-immune COBRA group and not in the Pre-immune Mock group. Robust IgG binding activity to WT H2, H5, H7, N1, and N2 and the chimeric proteins cH6/1 and cH7/3 was confirmed in COBRA-vaccinated mice, while only a few showed detectable levels of binding activity to the H5 and N2 WT antigens (Figure 4). In the Pre-immune COBRA group, three of the nine mice with detectable lung viral titers had no HAI activity and lower binding to the AH/13 H7 protein and cH7/3 protein (Table S1). In the Pre-immune Mock group, only three out of the nine mice had no detectable lung virus at 3, 4, and 6 DPI; they all had IgG binding to AH/13, and one of them had low detectable binding to cH7/3 (Table S2). No standard has been established to correlate protection with that conferred by vaccination with pre-pandemic influenza strains [36]. The majority of the mock-vaccinated mice survived without HAI activity. Most had only baseline titers to the AH/13 H7 strain, and only two mice had binding activity to cH7/3. Most mock-vaccinated mice showed binding activity to the SW/13 N2 protein (Figure 5 and Figure 6). It is possible that memory B cells responding to the NA component of Pan/99 H3N2 are being recalled and expanded after H7N9 challenge, since little to no H7-specific ASCs were present after the H7 challenge (Figure 7B). This is explained to some extent by our results in mock-vaccinated mice. Anti-NA antibodies could not prevent viral entry into host cells leading to high viral lung titers and extensive lung injury, but they did limit viral spread during infection. Anti-NA antibodies can inhibit the enzymatic activity of NA, preventing the cleavage of sialic acid residues and thus reducing the release of newly formed virions from the infected host cell. NA-inhibiting antibodies may cooperate with HA stalk-directed antibodies to mediate and enhance antibody-dependent cellular cytotoxicity (ADCC) by activating the Fc receptors and increasing the Fc-Fc receptor contact, thereby mediating the killing of infected cells [37]. Both the HA stalk and NA are subdominant antigenic regions. The contribution of both types of antibodies to ADCC function is unknown. Cross-reactivity between the N2 antigen from preimmunization and the N9 antigen from the challenge strain has not been confirmed [38].

Less body weight loss, a higher survival rate, and lower clinical scores were observed in the COBRA HA/NA-vaccinated group. COBRA-vaccinated mice also cleared the virus in the lungs faster than mock-vaccinated mice (Figure 2). An average HAI titer of 1:40 was measured against the H5 strain panel, and 1–2 log lower HAI titers were detected in the H2 and H7 panels (Table 1). Significantly higher WT antigen binding activity was observed, as well as binding to both group 1 and group 2 chimeric antigens (Figure 4 and Figure 5). Vaccinated mice displayed an IgG bias towards the IgG1 subclass, resulting in an IgG1/IgG2a ratio greater than 2, indicating that the pentavalent COBRA vaccine elicits a Th2-polarized immune response in pre-immunized DBA2/J mice (Figure 6). Prior to challenge, the total Ig antibody responses were mainly composed of IgG1 in the COBRA-vaccinated group, while no total Ig response was measured in the mock-vaccinated group. The IgG1, IgG2a, and IgG2b ASCs in the spleens were boosted by the challenge in both groups (Figure 7A). The number of IgG1 and IgG2 ASCs was significantly increased compared to the number of ASCs before challenge in the COBRA-vaccinated group (Figure 6). Before the challenge, only IgG1 ASCs were detectable by FluoroSpot in the COBRA-vaccinated group. However, IgG1, IgG2a, and IgG2b binding activity prior to the challenge was confirmed in both groups by ELISA. This disparity between the assays may be explained by the higher sensitivity of ELISA in detecting the endpoint titer or because a larger sample size was used for ELISA versus Fluorospot (ELISA: 16/group, FluoroSpot: 3/group; Figure 6 and Figure 7). In the COBRA-vaccinated group, GD/16 H7-specific IgG1 ASCs were significantly enhanced after the challenge and were significantly higher than in the mock-vaccinated group; it is likely that the H7 challenge strain stimulated the cross-reaction towards the common epitopes that both IAN8 and HK/17 can recognize (Figure 7B). Upon activation by HK/17, CD4+ T cells were stimulated and delivered the signal to the B cells, leading to the somatic hypermutation and differentiation of B cells into Th2 ASCs capable of secreting GD/16 H7-specific IgG1. Although the ASCs for total Ig antibodies were greatly increased in the mock-vaccinated group following the challenge, the GD/17 H7-specific IgG1 ASCs at 6DPI was still lower than 10 spots per million cells. A small number of spots were present in the SC/14 H5-specific ASCs before and after the H7 challenge, indicating no cross-reactivity between the H5 and H7 antigens (Figure 7C).

One limitation of this study is the dose of each HA and NA protein in the vaccine formulation. The apparent advantage of including NA antigens in a multivalent vaccine is to provide protection by additional mechanisms targeting one or more additional steps of infection. However, a potential disadvantage could be an altered immunodominance between the HA and NA antigens due to a less-than-optimal ratio of the proteins. This may redirect the primary immune response to subdominant antigenic domains rather than maximizing the breadth of immunity to the core vaccine component [39]. Another limitation is the lack of a suitable group 2 HA, so testing for the binding to cH7/3 cannot exclude the binding to the H7 head, which is one of the components in the vaccine formulation.

Among humans, individuals have variable pre-existing immunity to H1N1 and H3N2 viruses due to each person’s unique natural infection and/or vaccination history. Because the immune system imprints on the first influenza strain it is exposed to, people born at different times imprint on different influenza viruses that circulated during their childhood. Evaluating influenza vaccines in an immunologically pre-immune model is the new trend for both preclinical and clinical trials. The COBRA methodology has previously been assessed in an immunologically pre-immune setting in both mouse and ferret models [26]. The current study demonstrates that a COBRA HA/NA vaccine can elicit broad-reactive antibody response and protect pre-immune mice from disease severity and mortality.

The 1:40 HAI titer has been a criterion to assess the vaccine effectiveness against seasonal influenza viruses, indicating that 50% of the subjects are protected by a seasonal influenza vaccine [40]. However, the same HAI antibody level is not a good correlate of protection for pre-pandemic strains. The 1:40 cut-off is not a predictor of vaccine effectiveness, the vaccine antigen platform might also contribute to this difference, and an adjusted cut-off of 1:80 was confirmed to be more useful in predicting the protection against A/Anhui/1/2013 challenge when the mice were vaccinated with a VLP antigen [41]. In the previous study, we confirmed the effect of H1N1 and H3N2 pre-immunity on the subsequent vaccination, the antibody levels were significantly higher in vaccinated mice with pre-immunity than vaccinated mice without pre-immunity after the first shot, and this difference was no longer existed after the second shot of COBRA vaccination [26]. In this study, the pentavalent COBRA pre-pandemic vaccine was tested in a pre-immune mouse model. In addition, NA components were incorporated into the vaccine to provide additional protection by preventing newly formed virions from budding from the cell surface for further transmission. IgG activities against N1 and N2 were confirmed in the COBRA-vaccinated mice, but we did not perform a functional assay to measure the neuraminidase inhibition (Figure 4). Quantification of the IgG subclass is important to illustrate the contribution of pre-existing immunity and vaccination. This is the first time that ASCs have been evaluated in pre-pandemic vaccination with seasonal influenza pre-immunity; IgG1, IgG2a, and IgG2b ASCs were enumerated before and after the H7 infection. Surprisingly, the H7 challenge did not change the dominance of IgG1 ASCs, suggesting that the memory B cell pools reshaped by two COBRA HA and NA vaccinations could not be altered by future infection. Vaccination with such a vaccine could elicit robust serologic and cellular immune responses and will protect healthcare workers from occupational risk. A pentavalent pre-pandemic vaccine consisting of 1 μg of each antigen will reduce the production burden and costs and ultimately benefit pharmaceutical companies.

## Figures and Tables

**Table 1:** Serum was taken from COBRA-vaccinated mice on day 42 and tested for HAI activities. H2 (A or top), H5 (B or middle), and H7 (C or bottom) represent HAI from the pre-immune COBRA group. The data shown are mean ± SEM, n = 16 per group in each experiment. The log_2_ HAI titers were determined and presented in a heat map form. Color cells represent mice that had a Log2 HAI titer equal to or higher than 5.32. Cells without color represent mice that did not achieve a Log2 HAI titer equal to or higher than 5.32. The H2 panel consists of 6 VLPs: Clade 1 HAs (A/Mallard/Netherlands/13/2001/H2N9 or Ma/NL/01; A/Duck/Cambodia/419W12M3/2013/H2N2 or Duk/Cam/13), Clade 2 HAs (A/Taiwan/1/1964/H2N2 or TW/64, A/Duck/Hong Kong/273/1978/H2N2 or Duk/78), and Clade 3 HAs (A/Turkey/California/1797/2008/H2N8 or Tur/CA/08; A/Quail/Rhode Island/16-018622-1/2006/H2N2 or Qu/RI/16). The H5 panel consists of 5 viruses and 1 VLP: Clade 1 HA (A/Vietnam/1203/2004/H5N1 or VN/04), Clade 2.2 HA (A/Whopper swan/Mongolia/244/2005 or WS/05), Clade 2.3.2.1 HA (A/Hubei/1/2010/H5N1 or HB/10), Clade 2.3.4.4 (A/gyrfalcon/Washington/41088-6/2014/H5N8 or gy/Wa/14; A/Sichuan/26221/2014/H5N6 or SC/14), and Clade 2.3.4.4b HA (A/Astrakhan/32112/2020/H5N8 or Ast/20). The H7 panel consists of 6 VLPs: Eurasian-lineage HAs (A/Turkey/Italy/589/2000/H7N1 or Tur/Italy/00; A/Duck/Jiangxi/3230/2009/H7N9 or Duk/JX/09; A/Anhui/1/2013/H7N9 or AH/13; A/Hunan/2650/2016/H7N9 or HN/16; A/Guangdong/17SF003/2016/H7N9 or GD/16); and North American lineage HAs (A/bluewing teal/Ohio/658/2004/H7N3 or blu/OH/04).

A	H2 strain	ID	Log (2) HAI titer															
			11	12	13	14	15	21	22	23	24	25	31	33	34	35	41	42
	TW/64		2.32	2.32	2.32	3.32	2.32	2.32	5.32	3.32	5.32	3.32	2.32	2.32	2.32	2.32	2.32	2.32
	Duk/78		5.32	2.32	2.32	2.32	2.32	2.32	2.32	2.32	2.32	7.32	2.32	2.32	3.32	3.32	3.32	2.32
	Ma/NL/01		6.32	4.32	2.32	2.32	9.32	3.32	9.32	6.32	8.32	6.32	7.32	3.32	8.32	8.32	3.32	2.32
	Tur/CA/08		5.32	2.32	6.32	2.32	4.32	2.32	3.32	2.32	2.32	9.32	2.32	2.32	5.32	6.32	3.32	2.32
	Duk/Cam/13		5.32	3.32	2.32	2.32	7.32	2.32	7.32	4.32	6.32	6.32	5.32	2.32	6.32	7.32	2.32	2.32
	Qu/RI/16		5.32	2.32	2.32	2.32	2.32	2.32	2.32	2.32	2.32	7.32	2.32	2.32	3.32	3.32	2.32	2.32
B	H5 strain	ID	Log (2) HAI titer															
			11	12	13	14	15	21	22	23	24	25	31	33	34	35	41	42
	VN/04		5.32	5.32	5.32	6.32	5.32	5.32	6.32	6.32	4.32	4.32	4.32	5.32	5.32	5.32	5.32	2.32
	WS/05		5.32	5.32	5.32	5.32	6.32	5.32	6.32	6.32	6.32	6.32	4.32	6.32	7.32	7.32	5.32	2.32
	HB/10		3.32	3.32	5.32	2.32	7.32	3.32	5.32	6.32	2.32	5.32	4.32	2.32	7.32	7.32	4.32	3.32
	gy/Wa/14		2.32	4.32	2.32	2.32	2.32	2.32	5.32	6.32	2.32	7.32	5.32	5.32	8.32	8.32	4.32	2.32
	SC/14		2.32	2.32	2.32	2.32	2.32	2.32	2.32	5.32	2.32	4.32	3.32	2.32	5.32	5.32	2.32	2.32
	Ast/20		6.32	4.32	3.32	3.32	4.32	4.32	5.32	7.32	5.32	5.32	5.32	6.32	6.32	6.32	5.32	3.32
C	H7 strain	ID	Log (2) HAI titer															
			11	12	13	14	15	21	22	23	24	25	31	33	34	35	41	42
	Tur/Italy/00		3.32	2.32	2.32	2.32	5.32	2.32	2.32	3.32	2.32	9.32	2.32	2.32	4.32	4.32	3.32	2.32
	Blu/OH/04		5.32	2.32	2.32	2.32	4.32	2.32	3.32	2.32	2.32	8.32	2.32	2.32	5.32	5.32	3.32	2.32
	Duk/JX/09		6.32	2.32	2.32	2.32	5.32	2.32	3.32	3.32	2.32	9.32	2.32	2.32	5.32	5.32	3.32	2.32
	AH/13		9.32	2.32	2.32	3.32	6.32	2.32	3.32	4.32	5.32	11.32	3.32	2.32	6.32	7.32	6.32	2.32
	HN/16		7.32	2.32	3.32	3.32	6.32	3.32	6.32	3.32	4.32	7.32	3.32	2.32	4.32	4.32	7.32	2.32
	GD/16		6.32	2.32	2.32	2.32	2.32	2.32	3.32	2.32	3.32	8.32	2.32	2.32	4.32	4.32	4.32	2.32

## Data Availability

All data are included in the manuscript.

## Supplementary Materials

The following supporting information can be downloaded at: https://www.mdpi.com/article/10.3390/vaccines12070706/s1, Table S1: Correlates between viral loads and antibody response in COBRA group; Table S2: Correlates between viral loads and antibody response in Mock group.
