# Supplementary material for: A Computationally Optimized Broadly Reactive Hemagglutinin and Neuraminidase Vaccine Boosts Antibody-Secreting Cells and Induces a Robust Serological Response, Preventing Lung Damage in a Pre-Immune Model
Source: Vaccines (Basel). 2024 Jun 24;12(7):706. doi: 10.3390/vaccines12070706 (PMC11281495; doi:10.3390/vaccines12070706)
Supplement: Supplementary file 1 [file vaccines-12-00706-s001.zip › vaccines-2997810-supplementary.pdf]

A Computationally Optimized Broadly Reactive Hemagglutinin and Neuraminidase Vaccine Boosts Antibody Secreting Cells and Induces a Robust Serological Responses Preventing Lung Damage in A Pre-immune Model

Lung samples collecting day	Mouse ID	Viral titer-PFU/g	HAI-AH/13	ELISA-AH/13	ELISA-cH7/3
3DPI	11	-	640	40500	40500
	12	3.35×10^5	-	13500	4500
	13	-	-	40500	13500
4DPI	14	2.36×10^5	10	13500	4500
	15	-	80	40500	40500
	21	1.79×10^3	-	13500	13500
6DPI	22	-	20	40500	40500
	23	-	40	121500	40500
	24	-	2560	121500	40500

Table S1. Correlates between viral loads and antibody response in COBRA group. Lung viral titers, AH/13 H7 HAI titers, H7 HA-specific total IgG and group 2 HA stem-directed IgG titers were listed for the mice in Pre-immune COBRA vaccinated group sacrificed for sampling on 3, 4 and 6 days post-infection. ELISA in end-point GMT. "-", undetectable, viral titer < 100pfu/g.

Lung samples collecting day	Mouse ID	Viral titer-PFU/g	HAI-AH/13	ELISA-AH/13	ELISA-cH7/3
3DPI	51	1.94×10^6	-	1500	-
	52	-	-	500	-
	53	8.46×10^4	-	4500	-
4DPI	54	1.83×10^6	-	-	-
	61	1.36×10^4	-	-	-
	62	3.32×10^6	-	500	-
6DPI	63	-	-	500	-
	64	8.75×10^5	-	1500	-
	65	-	-	1500	500

Table S2. Correlates between viral loads and antibody response in Mock group. Lung viral titers, AH/13 H7 HAI titers, H7 HA-specific total IgG and group 2 HA stem-directed IgG titers were listed for the mice in Pre-immune COBRA vaccinated group sacrificed for sampling on 3, 4 and 6 days post-infection. ELISA in end-point GMT. "-", undetectable, viral titer < 100pfu/g, ELISA end-point GMT <500.
